# Cases with Observations, on Wry Neck; on the Reduction of Luxations of the Shoulder-Joint; on the Operation for Hare-Lip; on Cartilaginous Substances of the Knee-Joint; on Aneurism; on the Use of Stramonium; and on the Extraction of a Gum Catheter from the Bladder, by an Incision above the Pubes, under Singular Circumstances

**Published:** 1819-07-01

**Authors:** 


### VIII.

*Cases with Observations, on Wry Neck; on the Reduction of Luxations of the Shoulder-Joint; on the Operation for Hare-Lip; on cartilaginous Substances of the Knee-Joint; on Aneurism; on the Use of Stramonium; and on the Extraction of a Gum Catheter from the Bladder, by an Incision above the Pubes, under singular Circumstances.* By JOHN KIRBY, A. B. one of the Censors of the Royal College of Surgeons in Ireland; Surgeon to St. Peter's and St. Bridget's Hospital, and Lecturer on Anatomy and Surgery, Dublin. One Octavo Volume, pp. 166. London, 1819.

Mr. KIRBY's name is well known to our readers as a surgeon and anatomist, and we can assure them, that they will find few medical books of the same size and price as this, in which so much useful and important matter is contained. The work is full of practical facts, and is incapable of Analysis; we, therefore, recommend it to the perusal of every practitioner in surgery.

We cannot help recording here the following trait of Professional Philanthropy, if we may use the expression, which does honour to the present age.

St. Peter's and St. Bridget's Hospital, Peter Street, Dublin, was founded in the year 1810, at the sole expence of Mr. John Kirby, the author of the work here announced, by whose sole exertions it is still supported. It extends relief annually to *twelve thousand extern patients, and is capable of accommodating thirty intern patients.* A course of clinical lectures and reports are delivered every winter by Mr. Kirby, the revenue accruing from which, is expended on the establishment.

> "When energizing objects men pursue,
> What are the wonders which they will not do!"

---

